# Next of kin’s experiences of sudden and unexpected death from stroke - a study of narratives

**DOI:** 10.1186/1472-6955-12-13

**Published:** 2013-04-17

**Authors:** Åsa Rejnö, Ella Danielson, Linda Berg

**Affiliations:** 1 Institute of Health and Care Sciences, University of Gothenburg, The Sahlgrenska Academy, Box 45740530, Göteborg, Sweden; 2 The Stroke Unit, Kärnsjukhuset, Skaraborg Hospital, 54185, Skövde, Sweden; 3 Department of Health Sciences, Mid Sweden University, 83125, Östersund, Sweden

**Keywords:** Acute stroke, Carer, Death and dying, Narratives, Next of kin, Nursing, Sudden and unexpected

## Abstract

**Background:**

Death always evokes feelings in those close to the afflicted person. When death comes suddenly the time for preparation is minimal and the next of kin have to cope with the situation despite their own sorrow. The suddenness is found to be stressful for the next of kin and communication both with healthcare professionals and information about what has happened has been found helpful. The aim of this study was to illuminate the experiences of next of kin from the sudden and unexpected death of a relative from acute stroke.

**Methods:**

Data was collected over a 12-month period in 2009–2010. Twelve next of kin of patients cared for in stroke units who died suddenly and unexpectedly from stroke were interviewed using a narrative method. The narratives were analyzed using narrative thematic analysis.

**Results:**

Three themes emerged showing facets of next of kin’s experiences of a relative’s sudden and unexpected death from stroke: Divided feelings about the sudden and unexpected death; Perception of time and directed attention when keeping vigil; Contradictions and arbitrary memories when searching for understanding.

**Conclusions:**

To have to live in the aftermath of severe stroke is absolute horror in people’s imagination and death is seen as the lesser of two evils. The sudden and unexpected death totally pervades the next of kin’s life, directs their attention to the dying person and even causes them to forget themselves and their own needs, and leads to difficulties in information intake. It is a challenge for the healthcare professionals to be able to identify the individual needs of the next of kin in this situation.

## Background

A person’s sudden death is difficult for the next of kin. There is little time to prepare for it and one’s world is turned upside down. Research on next of kin’s experiences of end-of-life care has shown that the relative’s end of life evoked both feelings of guilt and shame in the next of kin [1] and also of being a devoted companion who knows the dying person best [2]. Next of kin report that communication and support from others, such as carers, is important and valuable to them in end-of-life care [2-4]. When next of kin are caregivers of a dying person, they experience it as challenging and their experiences can be expressed as a modified self, where their image of themselves is challenged by questioning their image of an ideal caregiver for a dying person, expose them to situations of close intimacy and by the mutual intertwined dependency between them and the dying family member [5].

Guidelines for stroke care, ranging from prevention to acute care to rehabilitation, can be found in Europe, USA, Canada, Australia, parts of South America and Asia [6]. According to national and international directives, persons afflicted by stroke should be cared for at special stroke care centers, often referred to as stroke units (SUs) [7-10]. The goal for SUs is primarily to give acute care and treatment but also to provide rehabilitative care [7]. However, when patients are severely afflicted by stroke, survival is not always possible. A stroke can cause anything from minor neurological deficits with practically no signs or symptoms to complete hemiparesis, or in severe cases lead to death. Stroke is the third leading cause of death in Sweden and Europe, and the fourth leading cause in USA [11-13]. Together with other cerebrovascular diseases, stroke constitutes the second leading cause of death worldwide [14]. Stroke is more common in older ages and 80% of persons afflicted by stroke in Sweden are above 65 years [11].

When people are afflicted by an acute and life-threatening illness such as an acute stroke and are cared for in the hospital, it is important that the next of kin are met and also get help from these carers in order to cope with this new life situation. The suddenness of the stroke onset has been found to have a stressful effect on the close relatives of those who survive their stroke because of their life situation’s dramatic change [15]. The close relatives were found to be in a fragile life situation, experiencing feelings of alienation, chaos, insecurity and of losing their foothold on life. In a study by Payne et al. [16], all patients with acute stroke, not only those likely to die, and their relatives were included. The initial uncertainty about whether the patient is going to survive or die and communication between patients, family members and healthcare professionals were factors central to a positive experience of care. Relatives also wanted to be included in a dialogue with professionals concerning decisions about care. Research from the acute stroke context show that nurses recognized the incident of stroke as being stressful to next of kin of dying patients also [17]. The nurses stated that the stroke comes like a bolt from the blue and puts the next of kin in a new situation in life. The illness comes suddenly and the available time proceeding death is limited. According to the nurses, the next of kin have to deal with both uncertainty and sorrow [17]. Moreover, there is an initial uncertainty about whether the patient is really going to die or if he/she might survive [16].

Sudden or unexpected death has mostly been studied from the perspective of acute and emergency care [18], intensive care [19] and cardiology settings [20] all of which have shown the importance of providing information to the next of kin. Research with stroke team members has shown that findings from other areas cannot simply be transferred to this context since stroke as illness influences the situation in specific ways which affects the situation, for example how ethical problems take a central role when death is a sudden and unexpected death [21]. In what way does the suddenness of acute stroke affect the next of kin and how do they experience the situation?

The aim of the study was to illuminate the experiences of next of kin from the sudden and unexpected death of a relative from acute stroke.

## Methods

### Design

Since the aim was to illuminate the next of kin’s experiences, a narrative method was used. “Narrative” as a concept has been defined in different manners [22,23]. Some researchers make a clear distinction between narratives and stories [24] but the term narrative is also often used interchangeably and synonymously with “story” for example within nursing research [22,25]. Generally it can be said that narratives are retroactive constructions – representations of experiences, things that have happened, including the narrators ordering of events into sequence and the effort to interpret and make something out of those events [22,26,27]. Narratives thus afford possibilities of understanding the past [22]. Polkinghorne [27] uses the terms narrative and story equivalent and argues that narrative can be seen as the process of making a story. We adopt his view in this article.

Narrative method is used in research within the care context, mainly to collect data, but also throughout analysis [28]. The method has been shown to be useful in illuminating people’s experiences of specific situations [15,25,29]. Narratives can thus be a valuable way of gaining knowledge that sheds light on the experiences of the next of kin in situations of sudden and unexpected death.

### Setting

Next of kin were recruited from the SUs of two associated hospitals in rural parts of western Sweden. Stroke care in Sweden is organized following the Helsingborg Declaration [8] as well as national guidelines [7,30]. These SUs carried out acute as well as rehabilitative stroke treatment and care in accordance with the SU directives, including teamwork between physicians specialized in stroke, nurses, enrolled nurses, occupational therapists and physiotherapists. The SUs admitted patients with suspected stroke around the clock all week. The two SUs had 30–32 beds at their disposal for an area with 242 000 habitants. The two SUs together cared for approximately 700 patients with stroke and 185 patients with transient ischemic attack (TIA) during 2010. The proportion of patients with stroke diagnosis dying during the acute phase of admittance was about 8%.

### Participants

From March 2009 to February 2010 next of kin of patients who were cared for at either of the recruiting SUs and who died from a sudden and unexpected stroke were asked to participate in the study. Next of kin of patients who had died due to the acute stroke during hospital stay and who agreed to participate were included. The acute phase is usually defined as within the first week. However, we set no fixed time limit. We viewed the stroke and the subsequent death as one unit regardless of the time span as long as death took place during hospital stay as a consequence of the acute stroke. Eleven next of kin to eight deceased patients were asked to participate. One declined for personal reasons. Two additional next of kin themselves asked to be included. Thus, twelve next of kin, four men and eight women aged 41–69 years (median 57 years) who were spouses, children and grandchildren, participated. The deceased persons, four men and four women aged 74–92 years (median 82 years) had been ill between three hours and twelve days before they died (Table 1).

**Table 1:** Showing next of kin sex, age and relation to the deceased and the deceased’s sex, age, length of stay before death and marital status

**Next of kin**			**The deceased**			
**Sex**	**Age**	**Relation to the deceased**	**Sex**	**Age**	**Length of stay before death**	**Marital status**
F	44	Daughter	F	81	3 hours	Widowed
M	49	Son	M	92	half a day	Widowed
F	69	Wife	M	74	2 days	Married
F	66	Daughter	F	90	2 days	Widowed
F	43	Granddaughter				
F	66	Wife	M	78	4 days	Married
F	66	Daughter	F	86	5 days	Widowed
M	53	Son	F	81	7 days	Widowed
M	60	Son				
M	63	Son				
F	55	Daughter	M	82	12 days	Married*
F	41	Daughter				

M male, F female.

* wife demented.

### Narrative

Interviews focusing on the participants’ narrative were used to collect data. The narratives were influenced by the stroke causing the sudden and unexpected death and by the context of care, the SU cf [22]. Thus the narratives do not speak for themselves, but require close interpretation and have to be understood in terms of the situation. The next of kin narrated their story, connecting the events from their relative’s sudden and unexpected death from stroke into a coherent story. In a strategic way, the next of kin chose and narrated events and thoughts perceived as significant and important with the goal of sharing their experiences with the researcher cf [22].

### Data collection – narrative interviews

Data was collected through narrative interviews which were performed, digitally recorded and transcribed as text by the first author. The interviews lasted 18–72 minutes (median 52 minutes) and were performed 15–44 days after death (median 27 days). To create a permissive climate, the interviews were carried out at places chosen by the participants. Thus, ten interviews took place in the participants’ homes and two in the first author’s office. The interviews were conducted as conversations focusing on the next of kin’s narrative. The opening question was: *“Could you please tell me about your experience of when your relative suddenly and unexpectedly had a stroke and died?”.* To allow a developed narrative providing detailed descriptions of their experiences of the situation, the researcher did not steer the interview, but instead let the next of kin follow their own trails in the narration cf [22]. Efforts were made to ensure that the narration was uninterrupted. The interviewer took the role of active listener and only follow-up questions such as: “*Could you tell me more about that?”* were used.

### Narrative analysis

The analysis started with the verbatim transcription of the interviews by the first author, immediately after data collection. Pitch and stressed words were marked and next of kin’s gestures were noted in the transcript, in order to preserve not only *what* was said but also *how* it was said [22,31]. The narratives were subjected to narrative thematic analysis [22] where the focus is on what is told in the next of kin’s narratives about their experiences from the onset of their relative’s stroke until the relative’s death. This analysis leads to themes in the same way as many other methods for analysis does and can be seen as a form of interpretative content analysis, with focus on the next of kin’s *individual* narratives. There is no theorizing from themes occurring in all the narratives, as is common in some qualitative research. In every interview, details and nuances in the narratives and what the next of kin narrated were paid attention to. The focus was on both the particular situation that the narrative told about and the context. From the analysis, case examples were chosen to illustrate the general patterns and the variations in the next of kin’s experiences of a relatives sudden and unexpected death cf [22].

### Ethical considerations

The study was approved by the Regional Ethics Review Board, Gothenburg, Sweden (dnr 615–08). Permission was also obtained from the director of each SU. The interviews followed guidelines in the Helsinki Declaration [32]; both orally and written information was supplied and verbal as well as written informed consent was obtained. Information was also given about the voluntarily nature of participation and the possibility of withdrawing without retaliation was also given. Confidential handling of the data ensures protection of the identity of both the participants and the deceased persons. The optimal time to approach and interview the next of kin was carefully considered. We assumed that the next of kin would be able to decide for themselves if time was right for them to participate in an interview. For this assumption we rely on research in death studies [33]. An effort was made to influence the next of kin in any way while they were deciding whether to participate in the study or not. Therefore, the nurse who had cared for the next of kin’s relative, not the researchers, approached them when they were leaving the SU after the relative’s death and asked if the next of kin were willing to take an information letter about the study. Approximately a fortnight after their relative’s death we got in contact with those next of kin who had accepted the letter. If they were positive we scheduled a time for the interview in accordance with their wishes. The interviewer was aware of the sensitive nature of the topic and paid extra attention to the reactions of the next of kin. At the least sign of discomfort, the next of kin were asked if they wanted to take a rest or cancel their participation. A counsellor at one of the hospitals was also available if necessary. None of the participants wanted to quit or needed to see the counsellor, but some of the interviews were paused.

## Results

The findings illuminate various facets of the next of kin’s experiences of having a relative who died a sudden and unexpected death from stroke. The findings are presented under the three themes which emerged from the thematic analysis. The names of the next of kin used in the quotations are fictitious, the dots marks pauses in the narration.

### Divided feelings about the sudden and unexpected death

The next of kin experienced a wide range of emotions from surprise over the event to sorrow about the imminent death and also relief that the severely afflicted relative would not survive.

It came so suddenly … I wasn’t at home, I was in town … I said goodbye to my husband when I left in the morning and … and he answered me and then … my daughter came and told me that dad was so ill … it came so suddenly --- it was tough … sad, because you knew it was near the end but at the same time maybe I thought it would be just as well if he passed away and didn’t have to lay like that but … at the same time it felt so good that he was alive too. We knew he was with us and it was hard telling which … I think the others thought so too, that it would be best if he was put to rest (Birgitta)

Death in this situation was viewed as the lesser of two evils and the next of kin felt no guilt over this feeling. The next of kin based this on their knowledge that the afflicted person would not have wanted to continue living in a vegetative state.

She wouldn’t have wanted to be a vegetable lying there and … only been … cared for and not be able to talk or do anything or … no it wouldn’t have been mum (Victoria)

To live on, severely handicapped by stroke, with no possibility of taking care of oneself, moving or communicating, was viewed as incredibly harsh, even cruel and a violation.

There are so many … different how it strikes … but if you look at some people then, well then it almost feels good that she could pass away … actually. One day be spry and alert and able to walk as far as anyone and the day after to be sitting in a wheelchair and not recognize visiting relatives … and with a diaper and everything, then it feels … well it can’t be easy (Anders)

It was considered disrespectful to the afflicted to just be kept alive. The next of kin thought it would mean a loss of dignity if the relative was unable to take part in life in the way the person would have wanted.

### Perception of time and directed attention when keeping vigil

Keeping vigil was experienced by the relatives as tedious and boring, even when the time between onset and death was short. Depending on how calm and peaceful the dying person was perceived to be, time could be experienced as passing slowly, as being like usual or as just disappearing. If the dying person was peaceful and breathing regularly the next of kin could sit quietly, look through the window, read a book or do a Sudoku. Holding the hand of the dying person to show they were there was experienced as calming and bestowing comfort, and the next of kin were pleased to do it. Being accompanied by someone they knew, such as a spouse or a sibling, eased the vigil. Those who kept vigil together could speak softly to each other or just sit quietly; the important thing was to not disturb the dying person.

Read some … and … some Sudoku and such too … and talked. I sat with my wife all the time, the same with my brothers, we were not alone. But you can’t sit talking all the time, it gets well … we read and … where there … well … noticed as soon … if there was any change (David)

The next of kin who kept vigil together mostly did not experience time as passing as slowly as those who were by themselves. Some next of kin tried to live as normally as possible despite keeping vigil and despite the imminent death. The next of kin felt that the carers encouraged them to carry on with life in the middle of the unusual situation that keeping vigil implies and believed that it would not disturb the dying person if the TV was on or if conversations took place in the room.

And my son said, the TV … we putted it on, because I said it might be disturbing if we have the TV and … sports on, - No it won’t because dad likes sports, he didn’t want to miss it, and so … so we had it on there then … we sat there as if everything was normal … in the evening I mean … the girls (enrolled nurses) they came there and turned him over and so on, I said it’s maybe disturbing – No it does no harm, they said … so it should be as usual then (Birgitta)

Abnormal breathing patterns, such as Cheyne-Stokes breathing, and mucous sounds were apparently the most stressful things for the next of kin. Caring acts that relieved the abnormal breathing sounds were experienced as positive. If the dying person’s breathing was affected the situation was perceived as troublesome for the dying person, then time was experienced as passing markedly slowly for the next of kin.

It was this breathing sounds we experienced as … hard … very hard especially during the night … it was hard to listen to (Maria)

The last few days she had very long … breathing pauses … there was a wall clock I sat and watched … sometimes it was … thirty seconds breathing pauses … and then she breathed for fifteen seconds and then it was thirty seconds again, it was … slow just sitting like that (Elisabeth)

If the next of kin had something other than the dying person to focus on, time was experienced to pass much less slowly. Above all, keeping vigil was a time when their attention was focused on the dying person, yet when there were periods of calm and quiet during the vigil, their attention turned towards themselves and their own body.

We sat about twelve hours every … we came in the morning and sat … until nine in the evening … we had to walk and get moving sometimes, you get stiff just sitting (Elisabeth)

When their attention was directed towards the dying person, the next of kin could totally repress experiences of their own physical body and its signals. The next of kin said they were reminded to take care of themselves and their basic need for food and rest, by the carers.

We went down to the café and had coffee and something to eat and so … because it was important, the nurse said: You must not forget to eat and drink. It is really important … that they reminded us about this. They told us to do so, reminded us when this happened … so … she invited us to do so (Birgitta)

This was appreciated since the next of kin thought they probably would not have eaten, drunk or rested otherwise. They described the difficulties in trying to take care of themselves since they could not stop thinking about the dying person even when they went home to get some proper sleep. If the telephone rang, they always were prepared that the call might be from the hospital to inform them that the dying person’s condition had deteriorated or even that the person had died.

### Contradictions and arbitrary memories when searching for understanding

It was also important to pay attention when receiving information and trying to understand the situation. Difficulties in taking in or remembering what had happened were occasionally apparent. The next of kin did not always remember what had happened. When their memory failed them the situation appeared blurred or unclear and there were blanks in their narratives.

But then I don’t know, because … well … it’s a bit fuzzy, everything to me, all this … just exactly when she died (Victoria)

There were contradictory statements in their narratives concerning their recollections. It was not unusual for the next of kin to make obviously inconsistent statements as in the following quotation where the next of kin says they never met a physician during the hospital stay and yet relates an episode involving one.

I never saw any doctor … after my mother had passed away and we sat with her … then a doctor came in … he just trudged in there (David)

The next of kin valued information and thought that it was necessary in order to understand the situation. Information made the situation intelligible and facilitated adjustment to the imminent death. While next of kin could experience that they had not received any information about what had happened to the dying person or about the cause of death, they were able to give detailed descriptions about the causes and the probable outcome of the event.

I wasn’t there so I never met the doctor but she had a clot, that was why it took twelve hours or something like that before you could like see it on the CT scan … this one was very powerful they said (David)

The next of kin themselves stated they had perhaps not taken in the given information and related this to being affected by the sudden and unexpected onset. It was as if they were in shock and any information given needed to be clear and precise.

Maybe the information was very clear yet … one doesn’t know that … but you don’t take it in (David)

This was especially true when receiving sad and serious notifications, namely, notifications that the next of kin did not want to receive, mostly concerning life and particularly death. The next of kin also talked about a feeling of uncertainty mainly regarding the outcome for the afflicted person. According to the next of kin, the carers should be able to give a clear answer about if, and in that case when, the afflicted person was going to die, but they also understood that no one can predict that with any certainty.

With that experience one might be able to know that … well like the doctor, that he could have predicted that … but at the same time I understand that one can never say that for sure (Erik)

The next of kin did not want to know that death was imminent, yet at the same time they wanted straight answers about what was to be expected. They did not want to be spared from the truth yet at the same time they did not want vulgarity and insensitivity. Clarity was said to be the absolutely most important thing as well as repetitions of the central message.

I think the doctor could have said” the damage is so widespread so I believe that … within six hours she will no longer be conscious … so I recommend you to … stay now … because it is during this time we can make contact with her” … if she had said more like that … we would have stayed then. It was exactly what she said although she said it … a little … well … by-the-by … and under those circumstances it needs to be hammered home. There were more carers saying that: You’re welcome to stay … because it is serious, they said. Although the message didn’t reach us (John)

Such contradictory statements could also concern the next of kin’s understanding of their experiences of contact with the dying person. They said they did not have any contact, yet on the other hand could also say they felt the dying person was aware of their presence.

My husband didn’t respond, but I still believe that you hear … maybe the last thing you lose is your hearing … my husband did a funny thing with his left hand so I am convinced that he heard (Inger)

## Discussion

By narrating their experiences, next of kin shed light on the situation and we provide possible interpretations and ways of viewing the sudden and unexpected death from stroke of a relative. First a short overall summarizing point is given and then the discussion follows in which each theme is going to be discussed separately. In some respects, the study illuminates the situation of sudden and unexpected death from stroke as being comparable to situations normally seen as being of more acute character, such as sudden cardiac death and trauma, related to cardiology- and ICU care. Thus, it presents a somewhat different view than the one normally thought of in connection with stroke where the emphasis is mostly on medical treatment, rehabilitation and remaining disabilities.

The next of kin’s *divided feelings about the sudden and unexpected death* were clearly visible in the narratives and showed how split the next of kin’s feelings can be. They were initially shocked by the incident. When they realized what sort of life their relative would have if he/she survived, and that this would not be the kind of life their relative would have wanted, they felt that death would be a relief and the best alternative. The findings concerning the initial shock, the unpreparedness for the difficult news and then acceptance of the relative’s imminent death are supported by research from ICU care [34]. This acceptance and even gratitude for the imminent death have also been seen in the context of cardiac care, once both the prospects and the conflicting feelings between wanting the loved one to live and death being a relief were considered [20]. Even in contexts where death was expected and seen as natural, as among older people, death could be seen as a relief based on the fact that the person’s suffering would come to an end [2].

The second theme *perception of time and directed attention when keeping vigil* illustrates the next of kin’s experiences and illuminates how experiences of time and attention are interdependent. Keeping vigil means taking on responsibility, making a commitment to the dying person, being there, being attentive to changes and keeping watch so that no harm will come to the loved one [35]. Commitment has previously been shown in the ICU context as advocacy, from the nurses’ perspective [36]. The nurses expressed it as a commitment to take on the responsibility to promote the welfare of another human being whose autonomy is threatened, which encourages the nurses to protect the patient. Advocacy has also been described from an end-of-life care context both from the perspective of the next of kin [37] but also from the nurse’s perspective regarding critical care for older patients [38]. In those studies, advocacy was not seen as commitment but as ensuring good care. Advocacy has also been found to be facilitated by commitment to professional codes of ethics [39]. This study’s findings suggest that the next of kin experience a commitment created by the suddenness and unexpectedness of the onset. The next of kin’s attention was focused while keeping vigil and the direction of it varied depending on the state of the dying relative. That the focus on the afflicted relative could be so strong that the next of kin forget to take care of themselves confirms findings from an ICU setting [34]. However, the finding that when the situation was quiet and peaceful, attention was directed from the relatives to the next of kin themselves was not seen in the ICU setting [34]. Thus, keeping vigil means being attentive and watching. When there is little to pay attention to during the vigil, the next of kin’s attention can focus on other things, such as their own bodily needs.

*Contradictions and arbitrary memories in the search for understanding* theme captured the next of kin’s contradictory way of experiencing the situation and the dependency on memory for interpreting experiences. The findings reveal that the next of kin were very concerned about information. The experience that their memory “behaved” arbitrarily and unreliably makes it difficult for others to know what is true and is not true in the narrative. This feeling might create uncertainty and put an unnecessary strain on the next of kin. The findings suggest that information makes the situation understandable and possible to cope with, but also that the next of kin experience difficulties in taking in the given information. Both giving and receiving information has been shown to be of central importance in previous research in the care sciences, not least, in the face of unexpected events in situations where patients both survive and die [20,40,41]. Contradictory statements were found in all narratives concerning information but also experiences and events that occurred during the care episodes. The contradictory way of experiencing situations of sudden and unexpected death is barely noted in previous research, possibly because it is thought to be common knowledge, but it is worth reflecting on the consequences this might have for the next of kin’s experiences and memories of the situation. Since memories are the only thing left from the time of the relative’s death, the way one remembers is crucial. When things seem contradictory, it is hard to fit memories together into a coherent whole. Inconsistencies give rise to conflicting memories of situations already loaded with feelings and might make a difficult situation even more difficult.

### Methodological considerations

The aim of the analysis is not only to summarize, but also to comment on and illuminate the meaning of the narratives in order to promote understanding and reveal the impact these situations have on people’s lives [42]. The analysis makes certain claims about how next of kin experience and understand sudden and unexpected death from stroke when relatives are afflicted and the meaning this has for them cf. [42]. The narrative form gave the participants the chance to express and share their thoughts, feelings and experiences but also to reflect on them. The knowledge possible to obtain from the narratives was limited by the intent and purpose the next of kin had with their narratives.

How sorrow affects person’s memories is also of importance for this study and the conclusions possible to draw from the next of kin’s narratives. Advice from the literature has not stated the right time to perform after-death interviews [33,43]. Williams and colleagues [33] concluded that the next of kin themselves must be trusted to know if the time is right for them to tell about the loved one’s death. Cook [44] states that the most important thing is that the researcher makes completely clear that the participant understand the meaning of participation. We strove to make absolutely sure that the next of kin fully understood the meaning of their participation. Research about the function of the memory for recalling, in relation to the aspect of time has also been performed [33,45]. There has been suggested that interviews performed within the first six months are most conducive for a narrative reconstruction [33]. Addington-Hall and McPherson [45] states that the memory may be biased by both frequency and saliency of an event in that salient events tend to be over-reported and less salient event tends to be under-reported. Moreover frequent events can become typical and reported as less salient and memory strategies may result in frequent events collapsing into a generic memory rather than in separate events [45]. These arguments taken together guided us when deciding on when to perform the interviews since we wanted to assure as good recollections as possible and at the same time be aware of the sensitive situation the next of kin were in.

The nurses at the SUs were instructed to hand out information letters about the study to the next of kin for every consecutive patient afflicted by sudden and unexpected death during the study period. It is unclear whether the nurses decided to exclude any next of kin for some reason, and this constitutes a weakness in the design. When the approached next of kin were telephoned and asked about their interest in participating in the study, only one declined. Additionally, two next of kin themselves took initiative and offered to participate. Therefore, one can assume that next of kin in this situation are highly motivated to share their experiences.

The researcher strove to create a permissive atmosphere in the interview situation so that the next of kin would feel comfortable expressing thoughts that could be seen as critical. Since narratives are created but also co-created by the narrator and the researcher, the researcher strove to allow the narrators to follow their own trails in their narrations and not to interfere unnecessarily.

Validity in narrative research can be divided into two parts; what the narrator narrates - the validity of the story; and the researchers’ narrative – the validity of the analysis cf [22]. The next of kin’s narratives were seen as true in that they constituted the narrated meaning of their experience. Validity within this analysis was achieved through the researchers’ commitment to the narrative thematic analysis process and sensitivity while interpreting cf [22]. The first author’s knowledge about the care context was of great help in this process since interpretations of the narratives could be made in light of this experience. It also made interpreting a difficult balancing act where focus needed to be kept strictly on the narrator’s intention with the narrative. The co-authors contributed valuable and critical views concerning the first author’s interpretations and the themes. Validity or trustworthiness in the study is enhanced by extensive quotations which aim to give the reader the possibility of judging for themselves the trustworthiness of the analysis. Since people express themselves in different ways and have more or less easy to narrate, quotes are chosen that are eloquent and illustrative. Therefore not all participants are quoted although their narratives might have been as informative rich of data.

The findings of this study agree with earlier findings within other contexts of end-of-life care, which also support its validity within the context of sudden and unexpected death from stroke cf [22]. The new knowledge generated in this study about how sudden and unexpected death is experienced by next of kin is applicable in all contexts where patients with acute stroke are cared for and not solely in SUs. In addition, these findings have the potential to be transferable to similar situations with occurrence of sudden and unexpected death in other care contexts and with patients of various ages.

## Conclusions

Sudden and unexpected death from stroke evokes a spectrum of emotions in the next of kin, because in people’s imagination, it is absolute horror to live in the aftermath of severe stroke. Because severe stroke is seen as a violation of the afflicted person’s dignity, death is seen as the lesser of two evils. The sudden and unexpected death pervades the next of kin’s daily life, since keeping vigil directs their attention to the dying person and even makes them forget themselves and their own needs. Furthermore, their memory betrays them, leaving blurs and blanks in their recollections of what occurred. This leads to difficulties, not least, in taking in information. It is a true challenge for healthcare professionals and requires a great deal of knowledge and sensitivity to be able to identify the individual needs of the next of kin in this horror-creating situation which totally occupies their life and thoughts at this time of grief and sorrow. Future studies on how health care professionals make use of next of kin’s narratives in health care practice would be of great value to further tailor individualized care in this context.

## Competing interests

The authors declare that they have no competing interests.

## Authors’ contributions

All three authors designed the study. The first author conducted the interviews, made the transcription of the interviews and the initial analysis of the interview transcripts. Each step of the analysis was then scrutinized and discussed by all three authors. The second and third author made critical revisions to the manuscript and all the authors read and approved the final manuscript.

## Pre-publication history

The pre-publication history for this paper can be accessed here:

http://www.biomedcentral.com/1472-6955/12/13/prepub
